# Enhanced attention precedes self-initiated locomotion in an electric fish

**DOI:** 10.1186/1471-2202-15-S1-P104

**Published:** 2014-07-21

**Authors:** James J Jun, Andre Longtin, Leonard Maler

**Affiliations:** 1 Department of Physics, University of Ottawa, Ottawa, ON, Canada, K1N 6N5; 2 Department of Cellular and Molecular Medicine, University of Ottawa, Ottawa, ON, Canada, K1H 8M5; 3 Centre for Neural Dynamics of Physics, University of Ottawa, Ottawa, ON, Canada, K1H 8M5

## 

Volition is generally considered as a defining human faculty; but the outcome of a voluntary decision can be predicted by brain activity even before subject’s conscious awareness [1], and similar phenomena are observed in many species. Preparatory neural activities for voluntary movements involve movement planning and decision making [2], and active movements accompany heightened attention [3]. We show that enhanced attention precedes self-initiated movements in an animal model that exhibits a readily observable and quantifiable sensory acquisition rate. In addition to demonstrating preparatory increases in sensory sampling, our results reveal close similarities between the sensory sampling and the neural activity suggest associated with voluntary decision making [4].

Cortical activity precedes self-initiated movements by several seconds in mammals; this observation has led into inquiries on the nature of volition [1]. Preparatory neural activity is known to be associated with decision making and movement planning [2]. Self-initiated locomotion has been linked to active sensing indicative of enhanced attention [3]; however, the precise temporal relationship between sensory acquisition and voluntary movement initiation has not been established. Based on long-term monitoring of sensory sampling activity that is readily observable in freely behaving pulse-type electric fish, we show that heightened sensory acquisition precedes spontaneous initiation of swimming. *Gymnotus* sp. revealed a bimodal distribution of electric organ discharge rate (EODR) demonstrating Down- and Up-states of sensory sampling and neural activity; movements only occurred during Up-states and Up-states were initiated before movement-onset. EODR during voluntary swimming initiation exhibited greater trial-to-trial variability than the sound-evoked increases in EODR. The sensory sampling variability increased before, and declined after voluntary movement onset as previously observed for the neural variability associated with decision-making in primates [4]. In contrast, stimulus onset quenched the sampling variability similar to that previously reported in neural variability [5]. Spontaneous movements occurred randomly without a characteristic timescale, and no significant temporal correlation was found between successive movement intervals.

## Conclusion

Using statistical analyses of spontaneous exploratory behaviors and associated preparatory sensory sampling increase, we conclude that electric fish exhibit all the established hallmarks of volition, and that voluntary behaviors in vertebrates may generally be preceded by increased sensory sampling. The dorsal telencephalon is required for spatial learning in teleosts and might initiate movement via its projections to the tectum [6]. Our results therefore suggest that comparative studies of the neural basis of volition may therefore be possible in pulse-type electric fish, given the substantial homologies between the telencephali of electric fish and mammals.
